# Increased Expression of AKT3 in Neuroendocrine Differentiated Prostate Cancer Cells Alters the Response Towards Anti-Androgen Treatment

**DOI:** 10.3390/cancers13030578

**Published:** 2021-02-02

**Authors:** Marc Wiesehöfer, Elena Dilara Czyrnik, Martin Spahn, Saskia Ting, Henning Reis, Jaroslaw Thomas Dankert, Gunther Wennemuth

**Affiliations:** 1 Department of Anatomy, University Duisburg-Essen, D-45147 Essen, Germany; marc.wiesehoefer@uk-essen.de (M.W.); elena.czyrnik@uk-essen.de (E.D.C.); jaroslaw.dankert@uk-essen.de (J.T.D.); 2 Department of Urology, Lindenhofspital Bern, CHE-3012 Bern, Switzerland; martin.spahn@hin.ch; 3 Institute of Urology, University Duisburg-Essen, D-45147 Essen, Germany; 4 Institute of Pathology, University Duisburg-Essen, D-45147 Essen, Germany; saskia.ting@uk-essen.de (S.T.); henning.reis@uk-essen.de (H.R.)

**Keywords:** prostate cancer, neuroendocrine differentiation, NE-like LNCaP cells, AKT3, miR-17 family

## Abstract

**Simple Summary:**

Metastatic castration-resistant prostate cancer is still untreatable, and patients have a very short life expectancy after diagnosis. One factor that makes metastatic castration-resistant prostate cancer so aggressive and difficult to treat is neuroendocrine differentiation of prostate carcinoma cells. We already showed that neuroendocrine differentiation of LNCaP cells results in increased AKT3 expression. The aim of this study was to demonstrate the role of AKT3 in neuroendocrine differentiation. Therefore, the previously obtained in vitro data were validated and extended to tissue from patient with neuroendocrine prostate cancer, where we show the presence of AKT3 in neuroendocrine cells. Furthermore, we demonstrate the oncogenic consequences of an increased AKT3 expression including inactivation of apoptotic proteins and a potential role of some miR-17 family members, negatively regulating AKT3, in neuroendocrine differentiation.

**Abstract:**

Patients with advanced prostate carcinoma are often treated with an androgen deprivation therapy but long-term treatment can result in a metastatic castration-resistant prostate cancer. This is a more aggressive, untreatable tumor recurrence often containing areas of neuroendocrine differentiated prostate cancer cells. Using an in vitro model of NE-like cancer cells, it could previously be shown that neuroendocrine differentiation of LNCaP cells leads to a strong deregulation of mRNA and miRNA expression. We observe elevated RNA and protein levels of AKT Serine/Threonine Kinase 3 (AKT3) in neuroendocrine-like LNCaP cells. We used prostate resections from patients with neuroendocrine prostate cancer to validate these results and detect a co-localization of neuroendocrine marker genes with AKT3. Analysis of downstream target genes FOXO3A and GSK3 strengthens the assumption AKT3 may play a role in neuroendocrine differentiation. Overexpression of AKT3 shows an increased survival rate of LNCaP cells after apoptosis induction, which in turn reflects the significance in vivo or for treatment. Furthermore, miR-17, −20b and −106b, which are decreased in neuroendocrine-like LNCaP cells, negatively regulate AKT3 biosynthesis. Our findings demonstrate AKT3 as a potential therapeutic target and diagnostic tool in advanced neuroendocrine prostate cancer and a new mRNA–miRNA interaction with a potential role in neuroendocrine differentiation of prostate cancer.

## 1. Introduction

Prostate carcinoma (PCa) is the second most diagnosed cancer type in men worldwide [1]. The development and progression of PCa is strongly dependent on androgen, making the androgen receptor (AR) one of the most important therapeutic targets in advanced and metastatic PCa. Therapeutic agents focus on the inhibition of androgen production or on blocking AR signaling [2,3]. Nevertheless, many patients relapse and develop metastatic castration-resistant PCa (mCRPC), which is androgen independent, more aggressive and without further treatment options [4]. Many different mechanisms switching from androgen dependent to androgen independent tumor growth are discussed, including enhanced AR expression, AR mutations or AR evasion through other signaling pathways. Another disadvantage of androgen deprivation therapy (ADT) is the induction of neuroendocrine transdifferentiation (NETD) of prostate cancer cells to neuroendocrine (NE)-like tumor cells (NETC) [5].

NE cells represent only a small number of cells in the prostatic epithelium and are androgen independent [6]. In addition, they secrete many hormones and peptides, such as chromogranin A (CHGA) and B, serotonin, bombesin or somatostatin, and regulate proliferation, differentiation and secretion of the prostatic epithelium [7]. NETCs share these characteristics and form castration resistant NE cell foci in PCa [8]. Uchida and colleagues demonstrated that foci of NETD are elevated in high-stage and high-grade prostatic tumors, leading to androgen-independent growth and progression of the tumor as well as supporting the invasion and metastasis of surrounding prostate cancer cells [9]. Based on these results, many studies suspect a correlation between NETD and poor clinical outcome [10]. However, the mechanism for androgen-independent proliferation is not fully understood yet; nevertheless, there are indications that activation of the PI3K/AKT pathway is essential for neuroendocrine differentiation of prostate cancer cells [11].

AKT Serine/Threonine Kinase (AKT) is a kinase with many different substrates which regulates a multitude of cellular mechanisms, including cell survival, proliferation and metabolism [12]. Overactivation of AKT promotes tumor progression and AKT is one of the most frequently hyperactivated kinases in human cancers [13]. Several studies demonstrate the oncogenic role of AKT in tumor progression [14,15]. AKT3 as one of three AKT family members seems to play an important role in promoting the development of malignant melanomas, triple negative breast cancer, colorectal cancer [16,17,18] and it is also expressed in prostate carcinoma [19]. Furthermore, findings of Nakatani and Le Page suggest an involvement of AKT3 in later stages of PCa [20,21]. Interestingly, genomic analyses reveal high alterations of AKT pathway genes in human neuroendocrine PCa samples [22]. We had previously shown an expression change of AKT3 mRNA after NETD of LNCaP cells in vitro using microarray analysis [23].

MiRNAs are known to have an influence on the development and progression of prostate carcinoma and are supposed to play a role during NETD [24,25]. They are able to inhibit protein biosynthesis post-transcriptionally by targeting specific sequences in the 3′-untranslated region (3′UTR) of mRNAs. MiRNA deregulation and function during PCa development and progression had already been extensively studied and a multitude of target genes could be identified [26]. However, only a few studies deal with the influence of miRNAs during NETD of prostate cancer cells.

Here we demonstrate the role of AKT3 in NE-like LNCaP cells as well as neuroendocrine prostate cancer and new mRNA-miRNA interactions.

## 2. Results

### 2.1. AKT3 Expression Is Increased in NE-Like LNCaP Cells

**Figure 1:**
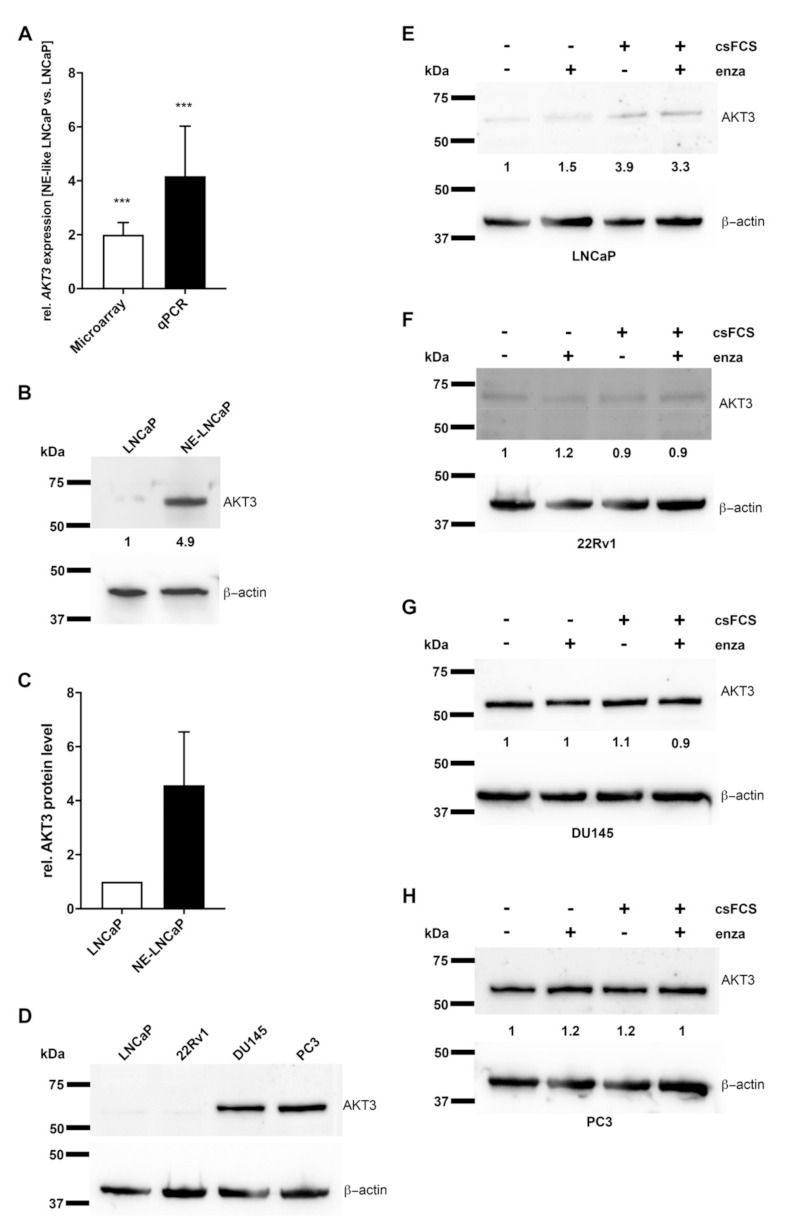
Expression of AKT3 is increased in neuroendocrine (NE)-like LNCaP cells and inhibited by androgen receptor (AR) signaling in prostate carcinoma (PCa) cells. (**A**) According to the results of microarray data from Dankert et al. 2018 (white bar; *p* = 0.0002) AKT3 expression was validated by qRT-PCR (black bar; *p* = 0.0007) confirming increased AKT3 expression in NE-LNCaP compared to normal LNCaP cells on mRNA level (*** *p* < 0.001). (**B**) Representative western blot analysis using anti-AKT3 mAb confirms induced AKT3 protein level in NE-like LNCaP cells. (**C**) Densitometrical quantification of western blot analyses represents the relative upregulation (4.5-fold) of AKT3 protein in NE-like LNCaP cells, determined in five independent experiments in relation to corresponding β-actin as loading control. (**D**) Western blot analysis of four PCa cell lines using anti-AKT3 mAb depicts different AKT3 expression in AR-positive and AR-negative PCa cell lines. PCa cell lines were treated with androgen deprivation or 10 µM enzalutamide followed by western blot assays for AKT3 expression. The treatments increased AKT3 protein expression in LNCaP cells (**E**), whereas AKT3 expression did not change in 22Rv1 (**F**), DU145 (**G**) and PC3 (**H**) cells.

NETD of AR expressing LNCaP cells was performed by cultivating LNCaP cells in androgen depleted medium for 14 days. Using microarray analysis, we had previously demonstrated a dramatic change in mRNA and miRNA expression after NETD of LNCaP cells in vitro [23], showing that AKT3 expression had significantly (*p* = 0.0002) increased 2-fold. To analyze if AKT3 plays a role in neuroendocrine differentiation of prostate carcinoma, we validated AKT3 expression in NE-like LNCaP cells compared to undifferentiated LNCaP cells in four independent differentiations by qRT-PCR (Figure 1A). The results show a strong accordance to former microarray results and reveal a significant (*p* = 0.0007) induction of AKT3 (4-fold). Subsequently, we analyzed the AKT3 protein level of neuroendocrine differentiated LNCaP cells in five independent experiments using western blot analysis. Figure 1B shows a representative western blot and the statistical evaluation (Figure 1C). In accordance to the results of AKT3 mRNA expression, AKT3 protein expression was also increased (on average 4.5-fold), suggesting a correlation between AR signaling and AKT3 expression.

### 2.2. AKT3 Protein Expression Negatively Correlates with AR Signaling

AKT3 protein level was examined by western blot analysis in two AR-positive, LNCaP and 22Rv1, as well as two AR-negative, DU145 and PC3, prostate cancer cell lines [27,28] to confirm a correlation between AR signaling and AKT3 expression. Figure 1D shows almost no AKT3 protein expression in LNCaP as well as in 22Rv1 cells, in contrast to DU145 and PC3 cells, which show high AKT3 protein expression, indicating a negative correlation between AR signaling and AKT3 expression. In addition, we treated the four PCa cell lines 14 days with enzalutamide, an AR inhibitor, androgen withdrawal, by culturing cells in charcoal stripped medium or a combination of androgen withdrawal and enzalutamide. Subsequently, the AKT3 protein level was analyzed using western blot analysis. Androgen withdrawal and the combination with enzalutamide led to a 3.3- to 3.9-fold increased AKT3 protein level in LNCaP cells (Figure 1E). In contrast, the AR-positive 22Rv1 cells were not affected by androgen withdrawal or enzalutamide treatment (Figure 1F). In addition to wild-type AR, 22Rv1 cells also express the AR splice variant AR-V7. This splice variant lacks the ligand binding domain, making the AR permanently active, leading to a persistent activation of the AR signaling pathway, and thus, the 22Rv1 cells are resistant to androgen deprivation or treatment with enzalutamide [29,30,31]. In consequence, the AR signaling pathway was not inhibited by the treatments, resulting in unaltered expression of AKT3. DU145 and PC3 cells do not express AR, which makes them also resistant to androgen withdrawal or enzalutamide treatment. Figure 1G (DU145) and Figure 1H (PC3) show no alteration in AKT3 protein expression after such treatments. Taken together, suppressed AR signaling leads to increased AKT3 expression in PCa cells and AKT3 protein expression seems to be directly inhibited by AR signaling. In consequence, androgen withdrawal resulting in NETD of LNCaP cells leads to evaluated AKT3 protein level in NE-like LNCaP cells and could play an important role in NETD of PCa.

### 2.3. Neuroendocrine Differentiated PCa Cells Express AKT3

To analyze AKT3 protein localization in neuroendocrine differentiated PCa, we collected tissue samples from patients with prostate cancer, performed HE staining and determined the Gleason Score. Neuroendocrine differentiation significantly occurs more frequent in advanced PCa. To identify samples with areas of neuroendocrine differentiation, the clinical markers CHGA and synaptophysin (SYP) were used for immunohistochemical staining. Representative stainings of five samples from patients with advanced PCa (Gleason Score: 4 + 5) and the corresponding neuroendocrine differentiated areas are shown in Figure S1. Under these conditions, we were able to compile a cohort of 15 patients with a corresponding Gleason Score containing areas of neuroendocrine differentiation (Table 1).

**Figure 2:**
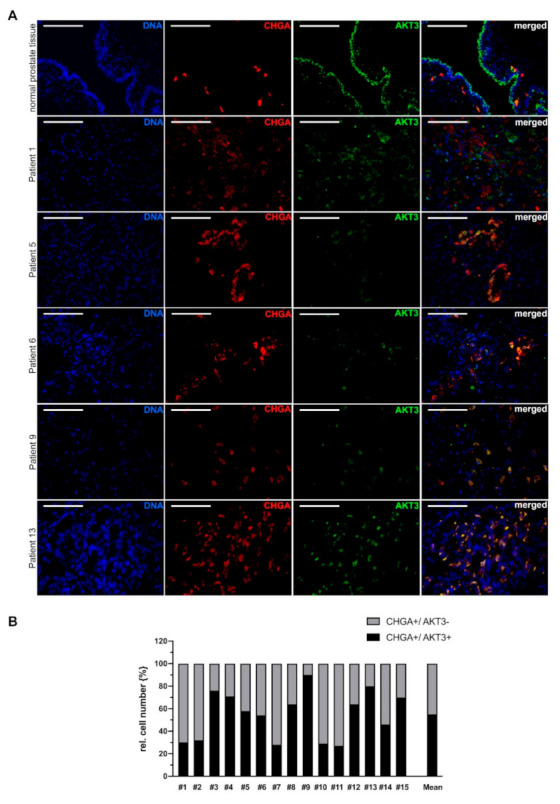
AKT3 colocalization with NE cell marker chromogranin A (CHGA) in advanced prostate cancer tissue. (**A**) Immunofluorescence double staining of normal prostate tissue and representative tissue samples from five patients with neuroendocrine differentiated prostate carcinoma showing CHGA-positive cells expressing AKT3 (DNA: blue, CHGA-positive NE-cells: red, AKT3: green, double positive cells: yellow; Magnification: 400×, scale bar: 100 µm). (**B**) Three areas per patient with neuroendocrine differentiated prostate cancer were documented and only samples with more than 20 neuroendocrine cells were analyzed. The number of CHGA-positive NE cells was normalized to 100% (grey bar). Graph shows statistical evaluation that demonstrates colocalization of CHGA and AKT3 (black bar) in approximately 55% of neuroendocrine cells.

To investigate whether neuroendocrine differentiated PCa cells express AKT3, immunofluorescence double staining was carried out. Tissue from gallbladder (Figure S2A) and small intestine (Figure S2B) were used to validate immunofluorescence staining with an unspecific mouse or rabbit serum instead of the primary antibody serving as control. Neuroendocrine cells were stained red with anti-CHGA or anti-SYP antibody, while cells expressing AKT3 were stained green. The first picture set of Figure 2A shows immunofluorescence double staining (CHGA, AKT3) of normal prostate tissue and demonstrates AKT3 expression in normal prostatic epithelium. Furthermore, we detected NE-cells which are AKT3 positive, suggesting a subpopulation of AKT3 expressing NE cells. Representative tissue samples from five different patients with neuroendocrine PCa indicate that many neuroendocrine cells also express AKT3 (Figure 2A). Statistical analysis of the images demonstrates that AKT3 is colocalized in approximately 55% of CHGA-positive cells (Figure 2B).

**Figure 3:**
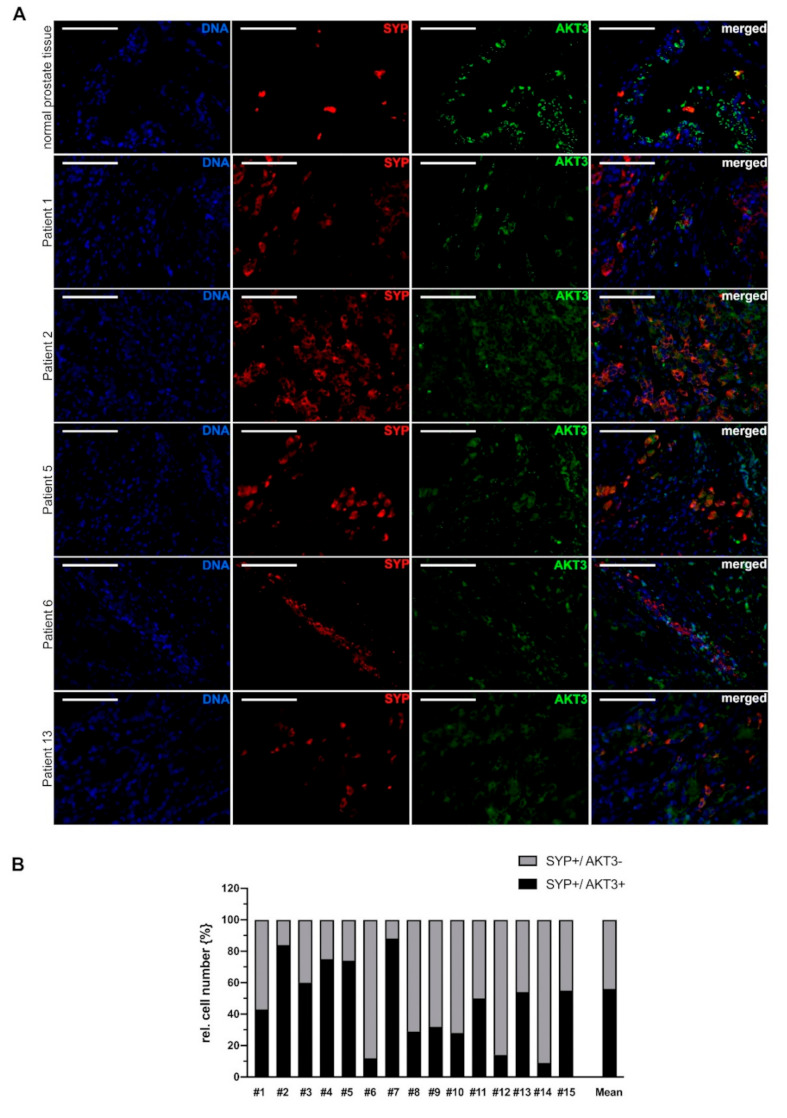
AKT3 colocalization with NE cell marker SYP in advanced prostate cancer tissue. (**A**) Immunofluorescence double staining of normal prostate tissue and representative tissue samples from 5 patients with neuroendocrine differentiated prostate carcinoma showing SYP-positive NE cells expressing AKT3. AKT3 frequently is colocalized with SYP in normal prostate tissue as well as tissue samples from patients with neuroendocrine PCa, indicating expression of AKT3 in NE cells (DNA: blue, CHGA-positive NE-cells: red, AKT3: green, double positive cells: yellow; Magnification: 400×, scale bar: 100 µm). (**B**) Three areas per patient with neuroendocrine-differentiated prostate cancer were documented and only samples with more than 20 neuroendocrine cells were analyzed. The number of SYP-positive NE cells was normalized to 100% (grey bar). Graph shows statistical evaluation that demonstrates colocalization of SYP and AKT3 (black bar) in approximately 55% of neuroendocrine cells.

These results were validated using synaptophysin as an additional marker for neuroendocrine cells. One picture set of normal prostate tissue and five representative picture sets from patients with neuroendocrine PCa double stained for SYP/AKT3 are shown in Figure 3A. According to the results of CHGA/AKT3 double staining, we detected a subpopulation of neuroendocrine cells which express AKT3. Figure 3B shows the statistical analysis where about 55% of SYP-positive neuroendocrine cells express AKT3.

It should be noted, that colocalization of various NE markers and AKT3 varies greatly. In PCA tissue from patient 5, for example, approximately 55% of CHGA positive cells are also AKT3 positive, whereas in the same patient, 75% of SYP positive cells are AKT3 positive. Taken together, our results suggest that a subpopulation of neuroendocrine cells express AKT3 in advanced prostate carcinoma.

### 2.4. AKT3 Signaling Pathway Is Activated in NE-Like LNCaP Cells

**Figure 4:**
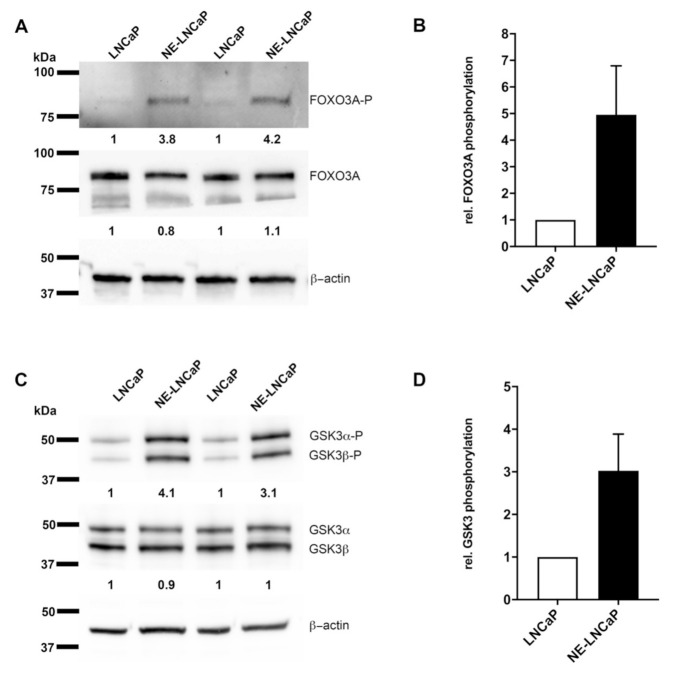
Downstream targets of AKT3 are phosphorylated in NE-like LNCaPs. AKT3 regulates the activity of Forkhead box O3 (FOXO3A) and Glykogen synthase kinase 3 (GSK3α/β) by phosphorylation. (**A**) LNCaP cells were cultivated in androgen free medium for 14 days. Representative western blot analysis of FOXO3A protein level and phosphorylation on Ser253 reveals no significant changes in protein level in comparison to phosphorylation. (**B**) Densitometrical quantification of four independent experiments prove a 5-fold increased phosphorylation of FOXO3A using β-actin as housekeeping gene. (**C**) Representative western blot analysis of GSK3α/β protein level and phosphorylation on Ser21/Ser9 demonstrates no modification of protein level, but an induction of phosphorylation. (**D**) Densitometrical quantification of four independent experiments show 3-fold increased phosphorylation on serine residues Ser21/Ser9 using β-actin as housekeeping gene.

Our results indicate that AKT3 may play a role in NE-like LNCaP cells as well as in neuroendocrine differentiated prostate cancer. Therefore, we examined whether the AKT3 signaling pathway is induced in NE-like LNCaP cells. AKT3 is a protein kinase, which phosphorylates serine and threonine residues and which hereby regulates the activity of different proteins as Forkhead box O3 (FOXO3A) or Glykogen synthase kinase 3 (GSK3α/β). For analysis of AKT3 downstream targets, we performed western blot analysis to determine the phosphorylation status of Ser253 (FOXO3A) and Ser21/ Ser9 (GSK3α/β). Neuroendocrine differentiation of LNCaP cells does not affect protein expression of FOXO3A, whereas analysis of phosphorylation at Ser253 shows an increase of phosphorylation (Figure 4A). Four independently performed experiments are depicted in Figure 4B and show a 5-fold elevated phosphorylation of FOXO3A at Ser253 using β-actin as loading control. Figure 4C,D illustrate that total protein level of GSK3 also is not affected by neuroendocrine differentiation of LNCaP cells, but the phosphorylation status of Ser21/Ser9 is 3-fold increased. These results indicate an induction of FOXO3A and GSK3α/β phosphorylation due to NETD of LNCaP cells.

### 2.5. Expression of AKT3 Has an Anti-Apoptotic Effect on AR-Positive LNCaP Cells

**Figure 5:**
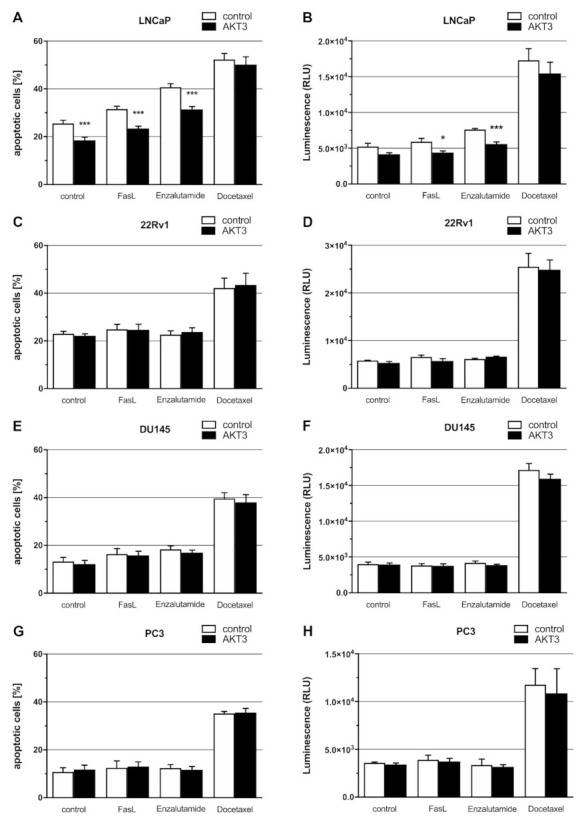
Effects of AKT3 expression on apoptosis of PCa cell lines were analyzed by Annexin V/propidium iodide (PI) staining and caspase 3/7 activity. Cells were transfected with control or AKT3 expression plasmid and apoptosis was induced by adding FasL (200 ng/mL), enzalutamide (10 µM) or docetaxel (20 nM) 24 h after transfection. Staining with Annexin V-FITC/ PI was performed 72 h post treatment and followed by flow cytometry analysis. Graphs show the mean ± SEM of four independent experiments. (**A**) Percentage of apoptotic cells decreased significantly (*** *p* < 0.001) after AKT3 overexpression in untreated LNCaP cells (*p* = 0.0008) as well as FasL (*p* = 0.0001) and enzalutamide (*p* = 0.0002) treated LNCaP cells. (**B**) Caspase 3/7 activity was measured 48 h after treatment by microplate reader. AKT3 overexpression in LNCaP cells has a slightly effect on caspase 3/7 activity. After induction of apoptosis with FasL (*p* = 0.0377; * *p* < 0.05) or enzalutamide (*p* = 0.0004; *** *p* < 0.001), AKT3 overexpression leads to a significant reduction of caspase 3/7 activity in LNCaP cells. AKT3 expression in 22Rv1, DU145 and PC3 cells has no effect on the relative proportion of apoptotic cells (**C**,**E**,**G**) as well as on caspase 3/7 activity (**D**,**F**,**H**).

Since we showed that the AKT3 signaling pathway is activated, we analyzed the effect of AKT3 on cellular properties such as proliferation, colony formation or apoptosis using an AKT3 expression vector. After transfection of PCa cells with an AKT3 expression vector, the AKT3 protein level of LNCaP cells were 13.6-fold (Figure S3A), 22Rv1 cells 16.8-fold (Figure S3B), DU145 cells 7.4-fold (Figure S3C) and PC3 cells 11.6-fold (Figure S3D) increased. Overexpression of AKT3 neither affected the cell number (Figure S3E) nor colony formation ability of LNCaP cells (Figure S3F). We further determined the effect of AKT3 on apoptosis using annexin V staining and caspase 3/7 activity assays. PCa cells were transfected with AKT3 expression plasmid and treated with 200 ng/mL Fas ligand (FasL), 10 µM enzalutamide or 20 nM docetaxel to induce apoptosis. Flow cytometry analysis of LNCaP cells stained with annexin V/ PI demonstrates that the percentage of apoptotic cells decreases after AKT3 overexpression (black bar) compared to the control (white bar), indicating an significant (*p* = 0.0008) anti-apoptotic effect of AKT3 on LNCaP cells. Treatment of LNCaP cells with FasL (7%), enzalutamide (15%) or docetaxel (25%) increases the percentage of apoptotic cells. Elevated AKT3 expression in LNCaP cells treated with FasL or enzalutamide leads to a significant (*p* = 0.0001 resp. 0.0002) 10% reduction of apoptotic cells (Figure 5A). These results suggest that AKT3 promotes cell survival in LNCaP cells. To analyze the molecular mechanism of AKT3 on apoptosis, we examined the caspase 3/7 activity. Figure 5B shows a slightly suppressed caspase 3/7 activity in LNCaP cells after enhanced AKT3 expression. In FasL treated cells, the caspase 3/7 activity is moderately elevated (1,2-fold) but AKT3 expression represses caspase 3/7 activity significantly (*p* = 0.0377) by about 35%. Enzalutamide treatment results in a 1.5-fold increased caspase 3/7 activity in LNCaP cells in comparison to untreated LNCaP cells, while elevated AKT3 level significantly (*p* = 0.0004) suppresses caspase 3/7 activity in LNCaP cells treated with enzalutamide to 65%. The treatment of docetaxel leads in both cases to a strong enhancement of caspase 3/7 activity (3.4-fold). The data demonstrate that upregulation of AKT3 in castration-sensitive LNCaP cells promotes survival by repressing caspase 3/7 activity. Apoptosis induction of 22Rv1 cells (Figure 5C) and elevated caspase 3/7 activity (Figure 5D) are only successful by treatment of 22Rv1 cells with docetaxel. The percentage of apoptotic cells increases to 40% while caspase 3/7 activity is 5-fold enhanced. AKT3 overexpression has no effect on cell survival of untreated or treated 22Rv1 cells. Due to the expression of AR splice variant AR-V7, 22Rv1 cells are castration-resistant [28,29]. The permanently active AR signaling protects cells from apoptosis and the activation of other survival stimulating pathways is not necessary. As well as 22Rv1 cells, DU145 and PC3 cells only respond to docetaxel treatment. The percentage of apoptotic DU145 cells elevates to 40% (Figure 5E) and caspase 3/7 activity increases 4-fold (Figure 5F). The percentage of apoptotic PC3 cells elevates to 35% (Figure 5G), while caspase 3/7 activity is 3.3-fold enhanced in PC3 cells after docetaxel treatment (Figure 5H). DU145 and PC3 cells are classified as castration-resistant PCa cell lines due to a lack of wildtype AR [27,28]. AKT3 overexpression has no effect on cell survival of untreated or treated DU145 and PC3 cells, but both cell lines stably express AKT3 (Figure 1D), indicating a certain amount of AKT3 expression is sufficient to support cell survival. Taken together, our results suggest that during androgen reduced conditions, castration-sensitive PCa cells undergo neuroendocrine differentiation associated with upregulation of AKT3 to become castration-resistant and to escape apoptosis.

### 2.6. Members of miR-17 Family Post-Transcriptionally Regulate AKT3 Biosynthesis

**Figure 6:**
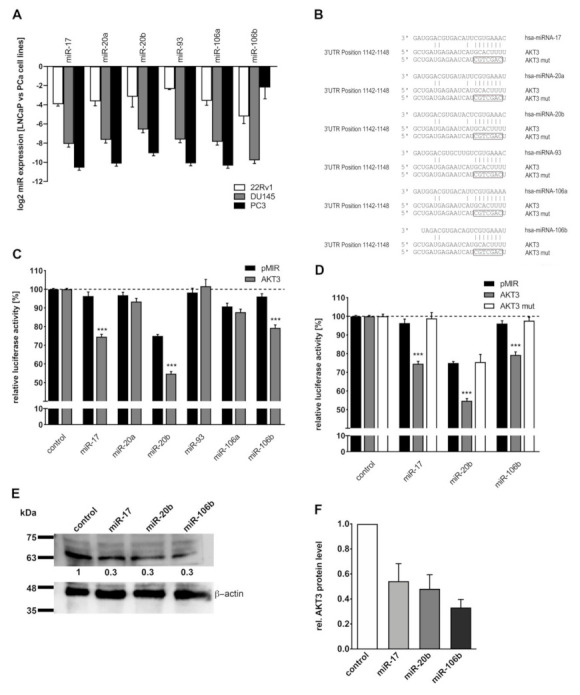
Post-transcriptional regulation of AKT3 protein expression by miR-17 family. (**A**) Expression of miR-17 family members in PCa cell lines was analyzed by qRT-PCR. MiR-17 family expression in 22Rv1, DU145 and PC3 cells was normalized to LNCaP cells. All members of miR-17 family are decreased (0.1-fold) in 22Rv1 cells whereas DU145 and PC3 cells do not exhibit any miR-17 family expression, except miR-106b in PC3 cells. (**B**) Predicted miR-17 family binding site in the 3′UTR of AKT3 mRNA and the mutated miRNA binding sites are shown. (**C**) MiRNA expression plasmids were cotransfected with control reporter plasmid (pMIR, black bars) or reporter gene construct containing AKT3 3′UTR (AKT3, grey bars). The luciferase activity of the control reporter gene plasmid coexpressed with control expression construct was set to 100 %. The graph shows the mean ± SEM of four independent experiments. MiR-17, -20b and -106b significantly reduce (20–25 %) luciferase activity of the reporter gene construct containing AKT3 3′UTR in comparison to the control (each *p* = 0.0001; *** *p* < 0.001). (**D**) Control reporter plasmid (black bars), reporter construct with wildtype seed sequence (grey bars) and the reporter construct with mutated seed sequence (white bars) was coexpressed with control, miR-17, -20b or -106b expression plasmids. After mutation of the seed sequence, the corresponding miRNAs are not able to reduce the luciferase activity. The graph displays the mean ± SEM of six independent experiments. (**E**) A representative western blot analysis for AKT3 of LNCaP cells which were transfected with control or miRNA expression plasmids, showing decreased (0.3-fold) AKT3 protein level after enhanced miR-17, -20b or -106b expression. (**F**) Densitometrical determination of three independent western blot analysis for AKT3 protein level after transfection with miRNAs using β-actin as loading control. MiR-17, -20b and -106b expression reduce (0.3–0.5-fold) the endogenous AKT3 protein expression. Uncropped Western blot images are provided in Figure S4.

Subsequently, miRNAs as a possible reason for enhanced AKT3 protein level in NE-like LNCaP cells were analyzed. Target gene analysis using TargetScan identified AKT3 as a putative target gene for the miR-17 family. We had previously shown a decreased expression of miR-17 family (on average 0.2-fold) in NE-like LNCaP cells, established by androgen withdrawal, in comparison to undifferentiated LNCaP cells [23], indicating a correlation between miR-17 family and AKT3 expression. Additionally, we compared the expression of miR-17 family members in LNCaP cells with 22Rv1 cells as well as DU145 and PC3 cells which stably express AKT3. In 22Rv1 cells, which express no AKT3, the miR-17 family expression is 0.1-fold lower than in LNCaP cells, whereas DU145 and PC3 cells do not express members of miR-17 family, except miR-106b in PC3 cells (0.25-fold) (Figure 6A). The putative miRNA target sites inside the 3′UTR of AKT3 are depicted in Figure 6B. A fragment of the AKT3 3′UTR containing the corresponding miRNA binding site was inserted into a luciferase reporter gene plasmid and luciferase reporter gene assays were performed. The reporter gene constructs were cotransfected with an expression vector for the respective miRNA. MiR-17, −20b and −106b significantly (each *p* = 0.0001) reduce luciferase activity of the AKT3 reporter gene construct by 20–25% (Figure 6C) whereas miR-20a, −93 and −106a do not repress the luciferase activity. The binding of the miRNAs to the predicted target site of the 3′UTR was confirmed by site directed mutagenesis of the corresponding miRNA seed sequence. The modified miRNA binding sites of AKT3 3′UTR are depicted in Figure 6B. Figure 6D shows an increase of luciferase activity to initial control level after mutation of the miRNA binding site inside AKT3 3′UTR, indicating a loss of miRNA binding. To investigate the regulatory abilities of the miR-17 family members on endogenous AKT3 biosynthesis, the respective miRNAs were ectopically expressed in LNCaP cells and AKT3 protein level was analyzed by western blot. A representative western blot analysis of reduced (0.3-fold) AKT3 protein level in LNCaP cells after overexpression of miR-17, −20b or −106b is depicted in Figure 6E. Figure 6F shows the mean of three independently performed western blot analyses after overexpressing miR-17, −20b and −106b, demonstrating that AKT3 is negatively regulated (0.3–0.5-fold) by particular members of the miR-17 family.

## 3. Discussion

The aim of this study was to discover the role of AKT3 in neuroendocrine PCa. In prostatic epithelium from healthy human donors, AKT3 is expressed in epithelial cells including normal NE-cells. We identified AKT3 expressing and non-expressing NE-cells in human prostatic epithelium. A possible explanation for the heterogenous expression pattern could be the presence of different NE cell subgroups. Several studies support this theory [32,33,34]. It is already well described that AKT3 is expressed in healthy prostate and prostate carcinoma [19], whereat Nakatani and colleagues gave a first hint that AKT3 could play a bigger role in advanced prostate cancer. They showed an elevated AKT3 expression in androgen-resistant in comparison to androgen-sensitive PCa cell lines [20]. Here, we confirmed these findings by demonstrating enhanced AKT3 protein expression in AR-negative PCa cell lines DU145 and PC3 cells compared to AR-positive LNCaP and 22Rv1 cells. Furthermore, suppression of AR signaling in castration-sensitive LNCaP cells, but not in castration-resistant 22Rv1, DU145 and PC3 cells, leads to an increased AKT3 expression, indicating a direct correlation between repressed AR signaling and enhanced AKT3 expression. Neuroendocrine differentiated LNCaP cells are also androgen-independent and we show an elevated expression of AKT3 mRNA and protein in NE-like LNCaP cells. Interestingly, Tai and colleagues demonstrated that PC3 cells also share common features with small cell neuroendocrine PCa (SCNC) like castration resistance and expression of NE markers [35]. Taken together, enhanced AKT3 expression in NE-like LNCaP cells and PC3 cells suggest a role of AKT3 in neuroendocrine differentiated CRPC. Another important finding was revealed by Le Page and colleagues showing a correlation of AKT3 expression in PCa with extra prostatic extension and hormone-refractory disease progression, suggesting an involvement of AKT3 in later stages of PCa [21]. Using two different markers for neuroendocrine differentiation, we observed an enhanced localization of AKT3 in areas of neuroendocrine PCa, which is much more common in advanced PCa with high Gleason Score [9,10]. This study cannot reach definite conclusions because specimens of 15 patients were analyzed only. The correlation between both NE markers and AKT3 varies in some patient specimens. However, several studies confirm that the clinical markers CHGA, SYP and NSE do not always have consistent specificity and sensitivity to neuendocrine cells [36,37], indicating multiple subgroups of NE cells differently expressing AKT3. Taken together, AKT3 seems to play a role in neuroendocrine differentiated, castration-resistant PCa.

Subsequently, the consequences of an increased AKT3 protein level in NE-like cancer cells were analyzed. Therefore, we determined the phosphorylation of two downstream target proteins FOXO3A and GSK3α/β after NETD of LNCaP cells. FOXO3A exhibits a stronger phosphorylation on Ser253 in NE-like LNCaP cells in comparison to untreated LNCaP cells, leading to inactivation and nuclear export of FOXO3A. Upon activation and translocation in nucleus, FOXO3A initiates apoptosis by inducing expression of apoptotic genes such as Fas ligand [38]. Additionally, Das and colleagues demonstrated that FOXO3A promotes apoptosis in castration resistant PCa [39]. Furthermore, a second downstream target of AKT3, GSK3α/β, was investigated which is also strongly phosphorylated in NE-like LNCaP cells. GSK3α/β is an important member in cell metabolism and is involved in glucose storage. AKT phosphorylates GSK3α/β at Ser21/Ser9, leading to its inactivation [40] and to increased survival rates of cells [41]. Subsequently, Mullholland and colleagues showed that GSK3 activation is frequently associated with advanced prostate cancer [42]. Taken together, AKT3 phosphorylates and inactivates FOXO3A and GSK3α/β, which could play a role in promoting apoptosis in NE-like LNCaP cells. Interestingly, FOXO3A inhibits cyclin D1 expression in colorectal cancer as well as breast cancer [43,44] whereas GSK3 phosphorylates cyclin D1 on Thr286, leading to ubiquitination and proteasomal degradation of cyclin D1 [45,46]. Therefore, AKT3 mediated inactivation of FOXO3A and GSK3 could lead to an increased cyclin D1 expression. Previously, we have already shown that cyclin D1 is elevated in neuroendocrine differentiated LNCaP cells [23]. Moreover, increased cyclin D1 is associated with increased cell growth and tumorigenicity in LNCaP cells [47] as well as in metastatic prostate cancer [48]. These signaling pathways significantly contribute to the characteristics of neuroendocrine differentiated PCa. To confirm these data, we analyzed the survival stimulating effect of AKT3 on castration-sensitive PCa cell line LNCaP and castration-resistant PCa cell lines 22Rv1, DU145 and PC3 by several in vitro assays. Our results confirm a survival stimulating effect of AKT3 expression by decreasing the percentage of apoptotic LNCaP cells as well as by repressing caspase 3/7 activity after apoptosis induction using Fas–Ligand or enzalutamide treatment. In contrast, elevated AKT3 expression has no anti-apoptotic effect on castration-resistant cells. 22Rv1 cells express AR splice variant AR-V7 which lack the ligand-binding site and makes it permanently active, stimulating survival [28,29]. DU145 and PC3 cells lacking AR signaling [27,28], but both cell lines endogenously express AKT3, suggesting AKT signaling is activated and sufficient to support cell survival. Additionally, it has been demonstrated by Lin and colleagues that AKT3 has an effect on PCa cell line proliferation [49] and AKT3 is overexpressed in malignant melanomas, supporting cell survival [16] as well as mediating apoptosis resistance in melanoma cells [50]. The negative regulation of FOXO3A and GSK3 and the anti-apoptotic effect of AKT3 on LNCaP cells support the theory, AKT3 plays a role in mediating apoptosis resistance in NETCs. Interestingly, the activation of PI3K/AKT signaling pathway via IGF1 or IL6 stimulates NETD in LNCaP cells [11,51] whereas inhibition of PI3K/AKT pathway using a PI3K inhibitor drives LNCaP cells into apoptosis [52]. The inhibition of AR signaling by androgen deprivation and activation of PI3K/AKT signaling by treatment with IL6 or IGF1 appears to be essential for NETD of PCa cells.

Previously, we could show that the miR-17 family is downregulated after NETD and ectopic expression of each member of the miR-17 family in LNCaP cells results in decreased cell proliferation, colony-forming ability and increased cell apoptosis [23]. Here, we demonstrate miR-17 family expression is higher in castration-sensitive compared to castration-resistant PCa cell lines and expression of AKT3 is post-transcriptionally regulated by members of the miR-17 family. According to this findings, increased protein biosynthesis of AKT3 after NETD of LNCaP cells may be caused by the decreased expression of miR-17 family. The miR-17 family expression was not analyzed in the corresponding clinical samples, because it was previously described that AR binding sites are located in miRNA flanking regions and analysis of castration-sensitive cells treated with synthetic androgen R1881 identified 16 upregulated miRNAs, including miR-106a, miR-17-5p, miR-20a, miR-20b, miR-19b, miR-93 [53]. Furthermore, AR directly regulates the expression of the miR-17-92a cluster, including miR-17, miR-20a, miR-18a, miR-19a/b and miR-92a in PCa. In AR-positive cells, a higher expression of miR-17-92a is observed compared to AR-negative cells and ectopic expression of AR could enhance the expression of miR-17-92a cluster in AR-negative prostate cancer cells while knockdown of AR decreased miR-17-92a expression in AR-positive cells [54]. According to this, inhibition of AR signaling, due to androgen withdrawal or enzalutamide treatment, would probably lead to a lower miR-17 family expression, resulting in increased AKT3 expression due to the direct miR-17 family mediated repression of AKT3 biosynthesis (Figure 6). All clinical samples show detectable neuroendocrine areas which are castration-resistant and AR-negative. Therefore, it is unlikely to find substantial expression of miR-17 family. Furthermore, Bhardwaj and colleagues were able to show that AKT3 is targeted by miR-29c [55] and expression of miR-29c is also stimulated by AR [53]. We found that miR-29c is also reduced (0.18-fold), similar to the miR-17 family, in NE-like LNCaP cells [23].

Taken together, our results suggest that neuroendocrine PCa may use other signaling pathways (PI3K/AKT) to compensate the loss of AR signaling to become androgen independent, to stimulate proliferation and to escape apoptosis. The knowledge about enhanced AKT3 activity and deregulated miRNAs which seem to regulate several members of PI3K/AKT pathway, could be used to develop a personalized diagnostic. Further studies should investigate whether the negative regulation of miR-17 family in NE-like LNCaP cells is also detectable in tissue or body fluids from patients with neuroendocrine differentiated prostate cancer. A further therapeutic approach is the combination of an androgen decreasing agent and a PI3K/AKT pathway inhibitor. A clinical phase II study was performed by de Bono and colleagues demonstrating an increased anti-tumor activity after combined treatment with abiraterone, an androgen-decreasing agent and ipatasertib, an AKT kinase inhibitor compared to treatment with abiraterone alone in patients with metastatic castration-resistant PCa [56]. A clinical phase III study is currently ongoing until end of 2023 [57].

## 4. Materials and Methods

### 4.1. Cell Lines

HEK293T cells (ATCC/LGC Standards GmbH, Wesel, Germany) were grown in DMEM (Thermo Fisher Scientific, Oberhausen, Germany) supplemented with 10% heat-inactivated FCS (Sigma Aldrich, Hamburg, Germany), Penicillin (100 U/mL) and Streptomycin (100 μg/mL). LNCaP, 22Rv1 and DU145 cells (ATCC/LGC Standards GmbH, Wesel, Germany) were cultivated in RPMI 1640 supplemented with 10% heat-inactivated FCS, l-Glutamin (1 mM final concentration), sodium pyruvate (1 mM final concentration), Penicillin (100 U/mL) and Streptomycin (100 μg/mL). PC3 cells (ATCC/LGC Standards GmbH, Wesel, Germany) were cultivated in Ham’s F-12K supplemented with 10% heat-inactivated FCS, l-Glutamin (1 mM final concentration), sodium pyruvate (1 mM final concentration), Penicillin (100 U/mL) and Streptomycin (100 μg/mL). For neuroendocrine differentiation, FCS was substituted by charcoal-stripped FCS (Sigma Aldrich, Hamburg, Germany) followed by LNCaP cultivation for 14 days. For Mycoplasma testing, cells were cultured on coverslips, fixed, mounted on slides with VECTASHIELD^®^ mounting medium containing DAPI (Vector Laboratories, Burlingame, CA, USA) and analyzed with a Leica DM4000 B microscope, Leica DFC340 FX camera and Leica Application Suite version 2.8.1 (Leica Microsystems, Wetzlar, Germany). All experiments were performed with mycoplasma-free cells. All cell lines have been authenticated using STR profiling within the last year.

### 4.2. RNA Extraction

Total RNA extraction from monolayer cells was performed with RNAmagic (Bio-Budget, Krefeld, Germany) in accordance to manufacturer’s instructions. MiRNA extraction from monolayer cells was carried out with miRNeasy Mini Kit (Qiagen, Hilden, Germany) in accordance to manufacturer’s instructions.

### 4.3. Quantitative Real-Time PCR

cDNA from mRNA was synthesized using High-Capacity cDNA Reverse Transcription Kit (Applied Biosystems, Darmstadt, Germany) while cDNA from miRNA was produced by miScript II RT Kit (Qiagen, Hilden, Germany). QRT-PCRs were performed using qTOWER^3^ (Analytik Jena, Jena, Germany), specific primers and 5× EvaGreen (R) QPCR-Mix II (ROX) (Bio-Budget, Krefeld, Germany). The thermal cycling conditions were as followed: 95 °C for 15 min followed by 45 cycles of 95 °C for 15 s, 58 °C for 30 s and 72 °C for 30 s. Melting curve analysis was performed for quality control. Evaluation of relative mRNA or miRNA expression was determined by ΔΔCt method using 18S rRNA (for mRNA) or 5.8S rRNA (for miRNA) as housekeeping gene. The oligonucleotides sequences are shown in Supplemental Table S1. For miRNA detection, oligonucleotide sequences are described elsewhere [23].

### 4.4. SDS-PAGE and Western Blotting

Cells were lysed with RIPA buffer (150 mM NaCl, 5 mM EDTA, 25 mM Tris, 0.1% SDS, 1% sodium deoxycholate, 1% NP-40) and the protein lysate were compounded with Laemmli buffer (62.5 mM Tris, 2% SDS, 25% glycerol, 0.01% bromophenol blue, 5% β-mercaptoethanol). Proteins were separated by electrophoresis using 8–16%-Mini-PROTEAN^®^ TGX Stain-Free™ Precast Gels and were transferred to Trans-Blot^®^ Turbo™ RTA Mini Nitrocellulose membrane (Bio-Rad, Düsseldorf, Germany). Immune detection of proteins was carried out using antibodies displayed in Supplemental Table S2 according to the manufacturer instructions. Proteins were visualized by Clarity™ Western ECL Substrate and ChemiDoc™ Touch Imaging System (Bio-Rad, Düsseldorf, Germany).

### 4.5. Human Prostate Specimens

PCa tissue from radical prostatectomy (RP) specimen (*n* = 142) was obtained from the Department of Urology at the Community Hospital Karlsruhe, Germany. Samples were paraffin-embedded. Fractions with >90% cancerous tissue were used. All patients were recruited from a well-characterized group of high-risk PCa patients of the EMPaCT tumor bank (European Multicenter Prostate Cancer Clinical and Translational Research Group) as described previously [58,59]. According to the high-risk PCa criteria established by D’Amico et al. [60], all patients had a preoperative/ initial serum prostate-specific antigen (PSA) of at least 20 µg/L. The study was approved by the local ethics committee (KEK Bern no. 128/2015).

### 4.6. Immunohistochemistry

Paraffin-embedded tissue blocks were cut at a thickness of 7 µm and slices were mounted on glass slides, deparaffinized with xylene and rehydrated in a descending alcohol series (100%, 96% and 70%). Slices were boiled in citrate buffer for antigen unmasking, were blocked for 60 min at room temperature with 1% BSA in PBS and were incubated overnight at 4 °C with rabbit anti-CHGA or anti-SYP antibody (1:200 in PBS with 0.5% BSA). After three washing steps with PBST, slices were incubated for 1 h at room temperature in PBS/0.5% BSA with diluted (1:200) secondary biotinylated swine anti-rabbit IgG. After washing three times with PBST, signal enhancement was achieved by Vectastain^®^ (Linaris, Wertheim-Bettingen, Germany) according to the manufacturer instructions. Immunoreaction was visualized with diaminobenzidine (DAB) (Sigma), followed by nuclear staining using hematoxylin solution (Thermo Fisher Scientific, Oberhausen, Germany). Finally, slices were dehydrated and mounted with Xylene Substitute Mountant (Thermo Fisher Scientific, Oberhausen, Germany). Slides were scanned using a Leica DM4000 B microscope, Leica DFC290 camera and analyzed using the Leica Application Suite version 2.8.1 (Leica Microsystems, Wetzlar, Germany).

### 4.7. Double Immunofluorescence

After rehydration, slices were treated with MaxBlock^®^ Autofluorescence Reducing Reagent Kit (Dianova, Hamburg, Germany). Antigens were unmasked with citrate buffer and slices were permeabilized using PBS/0.025% Triton X-100. After washing with PBS, slices were blocked for 60 min at room temperature with PBS/5% swine serum. Afterwards, slices were incubated overnight at 4 °C in PBS with 2% swine serum and diluted (1:100) rabbit anti-CHGA or anti-SYP antibody. After washing with PBS, slices were incubated for 1 h at room temperature with biotinylated swine anti-rabbit antibody diluted 1:100 in PBS. After two additional washing steps with PBS, slices were incubated for 1 h at room temperature with CY™3-conjugated streptavidin (Jackson Immuno Research, Ely, Cambridgeshire, UK) diluted 1:200 in PBS/0.5% swine serum, followed by two more washing steps with PBS. Sections were blocked for 30 min with PBS/1% BSA and incubated overnight at 4 °C with mouse anti-AKT3 antibody diluted 1:100 in PBS/0.5% BSA. After two rinses in PBS, slices were incubated for 1 h at room temperature with Alexa 488 coupled anti-mouse antibody, diluted 1:200 and DAPI (1:200) in 0.5% BSA/PBS, washed twice in PBS, treated with post detection conditioner and mounted with Fluoromount (Sigma-Aldrich, Munich, Germany). For signal detection, Leica DM4000 B microscope, Leica DFC340 FX camera and Leica Application Suite version 2.8.1 (Leica Microsystems, Wetzlar, Germany) was used.

### 4.8. Plasmids

Nucleotides 1609-2836 of the AKT3 mRNA (accession number: NM_005465.4) were amplified from human genomic DNA by PCR and inserted into pMIR-RNL-TK reporter plasmid (Ambion, Kaufungen, Germany). Mutagenesis of the predicted target site seed sequences of reporter constructs were performed by site directed mutagenesis and with sequence specific primers. The miRNA expression plasmids are described elsewhere [23]. AKT3 coding sequence (accession number: NM_005465.3) was amplified from human AKT3 gene cDNA clone plasmid (Hölzel Diagnostika Handels, Cologne, Germany) by PCR and inserted into pcDNA3.1 expression plasmid (Thermo Fisher Scientific, Oberhausen, Germany) for in vitro analysis. The primer sequences used for cloning and site directed mutagenesis are shown in Supplemental Table S1.

### 4.9. Transfection

HEK293T cells were transfected with Polyfect Transfection Reagent (Qiagen, Hilden, Germany) and LNCaP cells were transfected using jetPRIME (Polyplus transfection, SeÂlestat, France) in accordance to manufacturer’s instructions.

### 4.10. Cell Proliferation Assay

3 × 10^5^ LNCaP cells were seeded in 12-well plates, transfected with 2 μg expression plasmid and cultivated for 24–72 h. Cell numbers were measured on days 0 to 3 after transfection by detaching cells with trypsin and resuspending in 1 mL RPMI medium. For automatic determination of cell numbers, CASY 1 cell counter (Schärfe System, Reutlingen, Germany) was used.

### 4.11. Colony Formation Assay

1 × 10^6^ LNCaP cells were seeded in 6-well plates and transfected with 2 μg expression plasmid. Cells were detached by trypsin 24 h after transfection and seeded in 6-well plates (3000 cells/well). After culturing cells for 14 days, cultures were fixed for 5 min with methanol containing 12.5% acetic acid, stained with 0.5% crystal violet methanol solution for 25 min and washed with ddH_2_O. Wells were photographed using Fujifilm LAS-3000 gel documentation system (Kleve, Germany).

### 4.12. Flow Cytometric Determination of Apoptosis

Cells were seeded in 24-well plates and transfected with 0.5 μg expression plasmid. After 24 h, cells were treated with 200 ng/mL Fas ligand (FasL; Immunotools, Friesoythe, Germany), enzalutamide (10 µM final concentration; Hölzel Diagnostika, Cologne, Germany) in androgen-reduced medium or docetaxel (20 nM final concentration; Sigma-Aldrich, Munich, Germany) to induce apoptosis. Cells were collected three days after transfection and stained with Annexin V-FITC (Immunotools, Friesoythe, Germany) diluted 1:40 in 200 µl DMEM for 20 min on ice. Cells were washed with cold DMEM and stained with 200 μL propidium iodide solution diluted 1:200 in DMEM. The acquisition was directly performed utilizing a FACSCalibur System (Becton Dickinson, Franklin Lakes, NJ, USA) and analyzed by Cell Quest Pro™ Version 6 (Becton Dickinson, Franklin Lakes, NJ, USA).

### 4.13. Caspase 3/7 Activity Assay

Cells were seeded in 96-well plates and transfected with 0.1 μg expression plasmid. After 24 h, cells were treated with 200 ng/mL Fas ligand (FasL), enzalutamide (10 µM final concentration) in androgen-reduced medium or docetaxel (20 nM final concentration). To measure caspase activity the Caspase-Glo^®^ 3/7 Assay System (Promega, Mannheim, Germany) was used in accordance to manufacturer’s instructions. Determination of luciferase activity was carried out using FlexStation3 microplate reader (Molecular Devices, Biberach an der Riß, Germany).

### 4.14. Target Gene Prediction

MiRNA target gene prediction was carried out using TargetScan (release 7.1; http://www.targetscan.org/)

### 4.15. Dual-Luciferase Assay

2 × 10^5^ HEK293T were seeded in 24-well plates and were transfected with 0.8 μg of expression plasmid and 0.2 μg reporter plasmid after 24 h. Luciferase reporter assays were performed 48 h after transfection using the Dual-Luciferase Reporter Assay System in accordance to manufacturer’s instructions (Promega, Mannheim, Germany).

### 4.16. Data Analysis and Statistical Methods

Statistical evaluation was performed with GraphPad Prism 7 (Statcon GmbH, Witzenhausen, Germany). All statistical tests were performed as two-sided Student’s t-test and *p* values of <0.05 were distinguished as significant. Densitometrical analysis of western blots and colony formation assays were quantified by ImageJ 1.48v (National Institute of Health, Bethesda, USA). For statistical evaluation of AKT3 positive NE cells in immunofluorescence double staining from patients with neuroendocrine PCa, three areas of a patient sample were documented and more than 20 neuroendocrine cells were analyzed for AKT3 staining.

## 5. Conclusions

Neuroendocrine differentiated prostate carcinoma use other signaling pathways like PI3K/AKT to become androgen independent and stimulate tumor progression. Here we demonstrated the role of AKT3, a member of the AKT family in neuroendocrine prostate cancer and the deregulation of AKT3 by miR-17 family members. Our basic functional findings support the predominant studies that PI3K/AKT activity is necessary for neuroendocrine prostate cancer. The involvement of AKT3 in neuroendocrine differentiated PCa could be used to develop a new treatment, combining an androgen decreasing agent and an AKT inhibitor, which may enhance the health condition of patients with metastatic castration-resistant PCa. Further, the suggested regulatory role of miR-17 family on PI3K/AKT pathway members and the miR expression in samples from patients with mCRPC should be particularly studied, to establish a new simplified method for the diagnosis of the mCRPC.

## Figures and Tables

**Table 1:** Patient data (*n* = 15).

Patient	Age (Years)	Serum PSA (ng/mL)	pT-Stage	Gleason	GG	pN+	Adj. ADT
1	69	31.00	pT3a	3 + 4	2	yes	yes
2	72	32.00	pT3b	4 + 3	3	no	yes
3	65	83.70	pT4	3 + 4	2	no	yes
4	73	50.10	pT4	4 + 5	5	no	yes
5	62	23.40	pT3b	3 + 3	1	no	yes
6	72	23.92	pT3a	3 + 4	2	no	yes
7	63	22.61	pT4	4 + 3	3	yes	yes
8	73	32.80	pT3b	4 + 4	4	yes	yes
9	77	58.00	pT3b	3 + 4	2	yes	no
10	76	33.50	pT3b	4 + 3	3	no	yes
11	63	25.41	pT3b	3 + 4	2	yes	yes
12	70	48.35	pT3a	3 + 3	1	yes	yes
13	67	153.00	pT3a	3 + 4	2	yes	yes
14	56	42.70	pT3a	3 + 4	2	no	yes
15	77	110.00	pT3b	5 + 4	5	no	yes

Abbreviations: PSA, prostate-specific antigen; pT stage, pathologic stage after prostatectomy; GG, Grade group; pN, pathological proven lymph node metastasis; Adj. ADT, Adjuvant androgen deprivation therapy.

## Data Availability

All data generated or analysed during this study are included in this published article (and its supplementary information files).

## Supplementary Materials

The following are available online at https://www.mdpi.com/2072-6694/13/3/578/s1. Figure S1: Preparation of a patient cohort with neuroendocrine differentiated PCa. To identify neuroendocrine differentiated areas in patients with advanced prostate cancer (Gleason Score: ±3), tissue samples were HE and immunohistochemical stained with CHGA or SYP as clinical marker for neuroendocrine differentiation. Five representative samples from patients with high Gleason Score and CHGA as well as SYP positive areas are shown (Magnification: 100×, scale bar: 400 µm). Figure S2: Establishment of antibodies used in immunofluorescence double staining. Immunofluorescence staining of human gall bladder (A) and small intestine (B) was used as antibody control. Immunostaining of DNA is shown in blue (DAPI). The anti-AKT3 mAb (clone L47B1) shows AKT3 localization (green) in epithelial gall bladder cells, whereas anti-CHGA antibody detects CHGA (red) and anti-SYP antibody detects SYP (red) in neuroendocrine cells inside the epithelium of jejunum (Magnification: 400×, scale bar: 100 µm). Figure S3: Expression of AKT3 after transfection of PCa cells with AKT3 expression vector was validated on protein level by western blot analysis with anti-AKT3 mAb using β-actin as loading control (A–D). The results show increased AKT3 protein expression in LNCaP cells 13.6-fold (A), 22Rv1 cells 16.8-fold (B), DU145 cells 7.4-fold (C) and PC3 cells 11.6-fold (D). Effects of AKT3 expression on cellular behavior. Effects of AKT3 expression on cell growth and colony formation on LNCaP cells were analyzed after transfection of LNCaP cells with control or AKT3 expression plasmid. Cell number was determined every 24 h after transfection in four independent experiments (E). Colony forming ability was evaluated 14 days after transfection, by fixing and staining colonies with crystal violet and densitometrical analysis. Upper graph shows the mean ± SEM of four independent experiments, lower picture depicts one representative result (F). AKT3 induction has no impact on proliferation or colony forming capacity of LNCaP cells. Figure S4: Uncropped Western blot images. Table S1: Oligonucleotides sequences. Table S2: Antibodies.
