# Supplementary material for: Increased Expression of AKT3 in Neuroendocrine Differentiated Prostate Cancer Cells Alters the Response Towards Anti-Androgen Treatment
Source: Cancers (Basel). 2021 Feb 2;13(3):578. doi: 10.3390/cancers13030578 (PMC7867287; doi:10.3390/cancers13030578)
Supplement: Supplementary file 1 [file cancers-13-00578-s001.zip › cancers-1085417-supplementary-update/cancers-1085417-supplementary.docx]

Increased Expression of AKT3 in Neuroendocrine
Differentiated Prostate Cancer Cells Alters the Response
Towards Anti-Androgen Treatment

Marc Wiesehöfer, Elena Dilara Czyrnik, Martin Spahn, Saskia Ting, Henning Reis,
Jaroslaw Thomas Dankert and Gunther Wennemuth


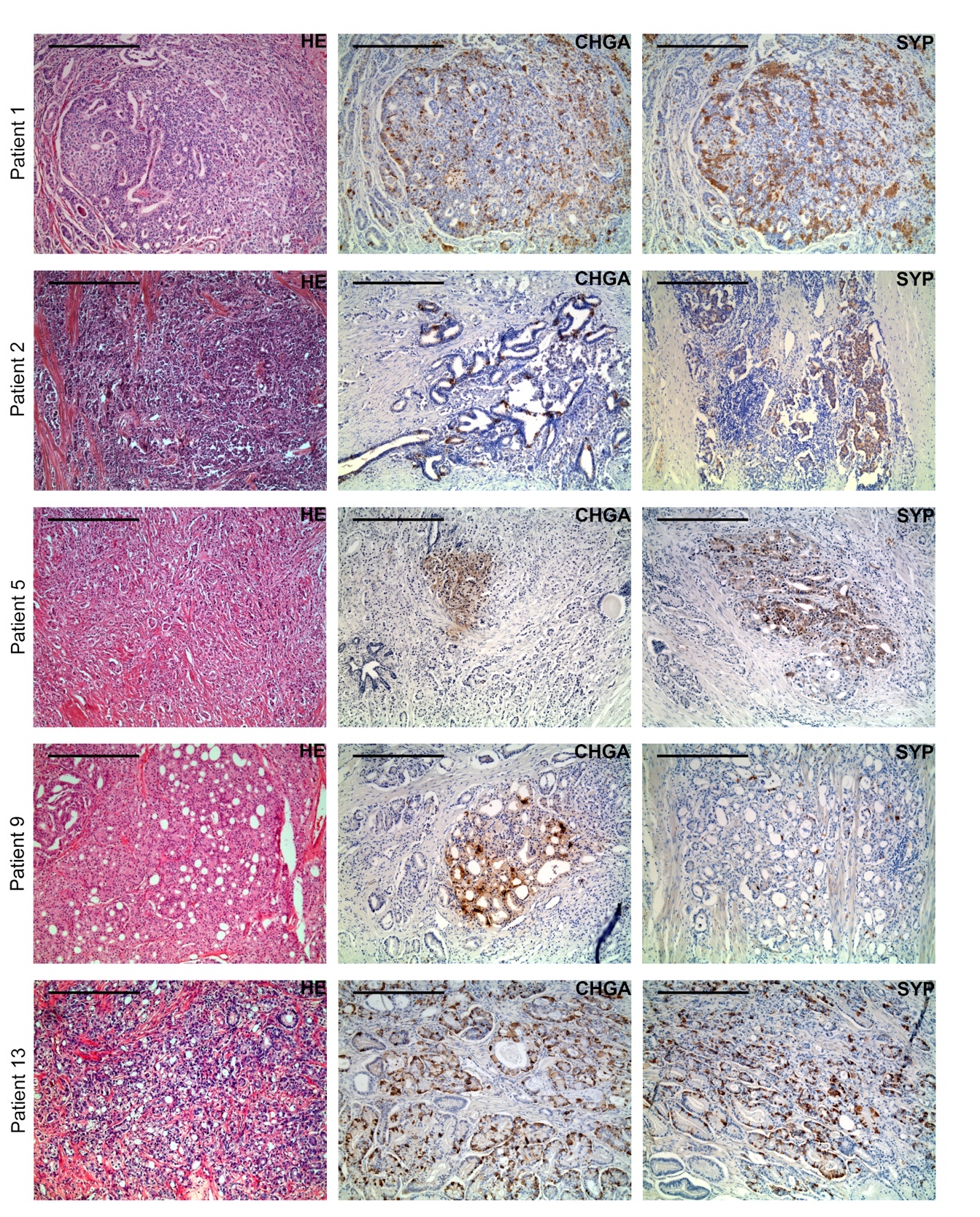


**Figure S1.** Preparation of a patient cohort with neuroendocrine differentiated PCa. To identify neuroendocrine differentiated areas in patients with advanced prostate cancer (Gleason Score: ≥4), tissue samples were HE and immunohistochemical stained with CHGA or SYP as clinical marker for neuroendocrine differentiation. Five representative samples from patients with high Gleason Score and CHGA as well as SYP positive areas are shown (Magnification: 100×, scale bar: 400 µm).


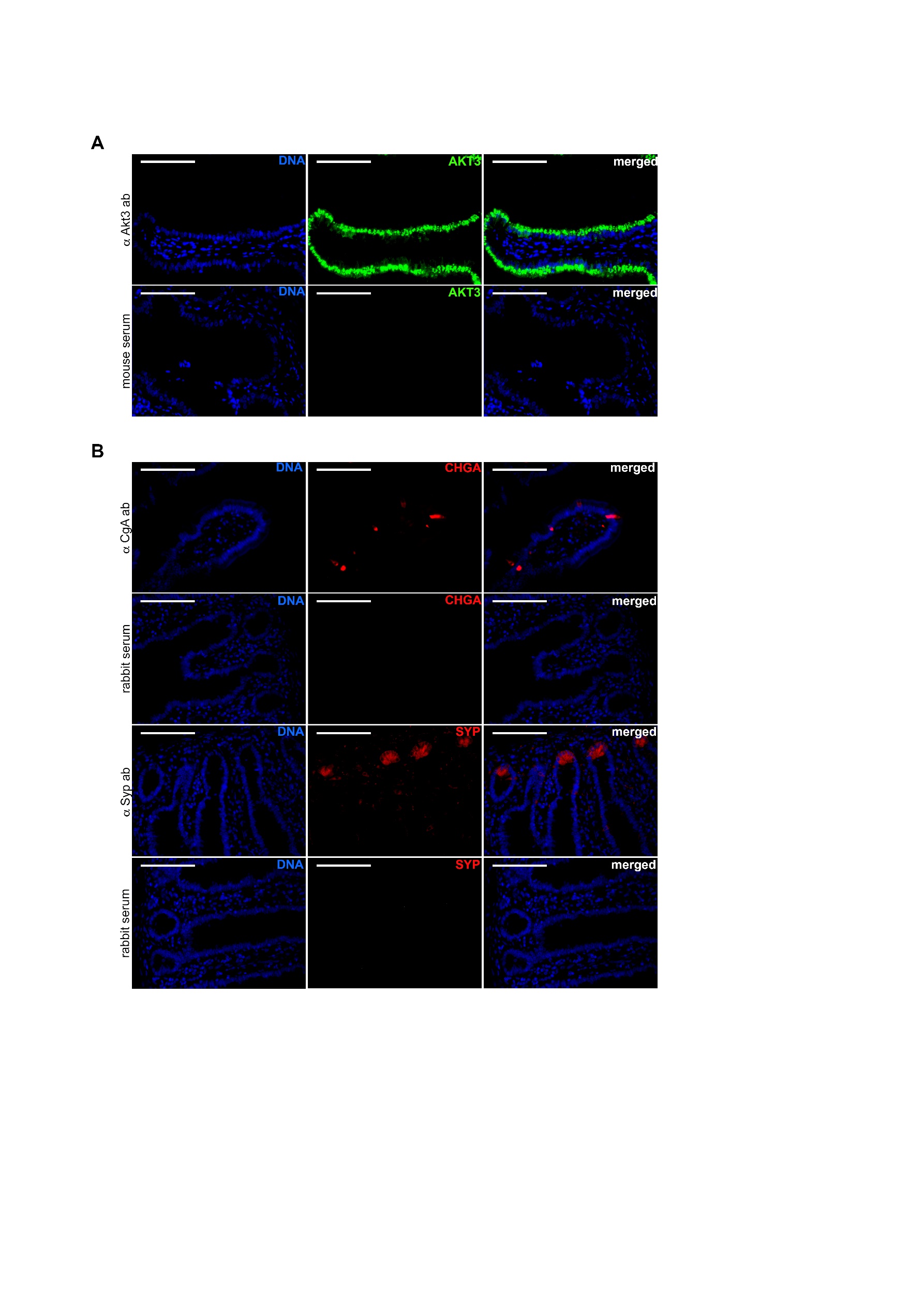


**Figure S2.** Establishment of antibodies used in immunofluorescence double staining. Immunofluorescence staining of human gall bladder (**A**) and small intestine (**B**) was used as antibody control. Immunostaining of DNA is shown in blue (DAPI). The anti-AKT3 mAb (clone L47B1) shows AKT3 localization (green) in epithelial gall bladder cells, whereas anti-CHGA antibody detects CHGA (red) and anti-SYP antibody detects SYP (red) in neuroendocrine cells inside the epithelium of jejunum (Magnification: 400×, scale bar: 100 µm).


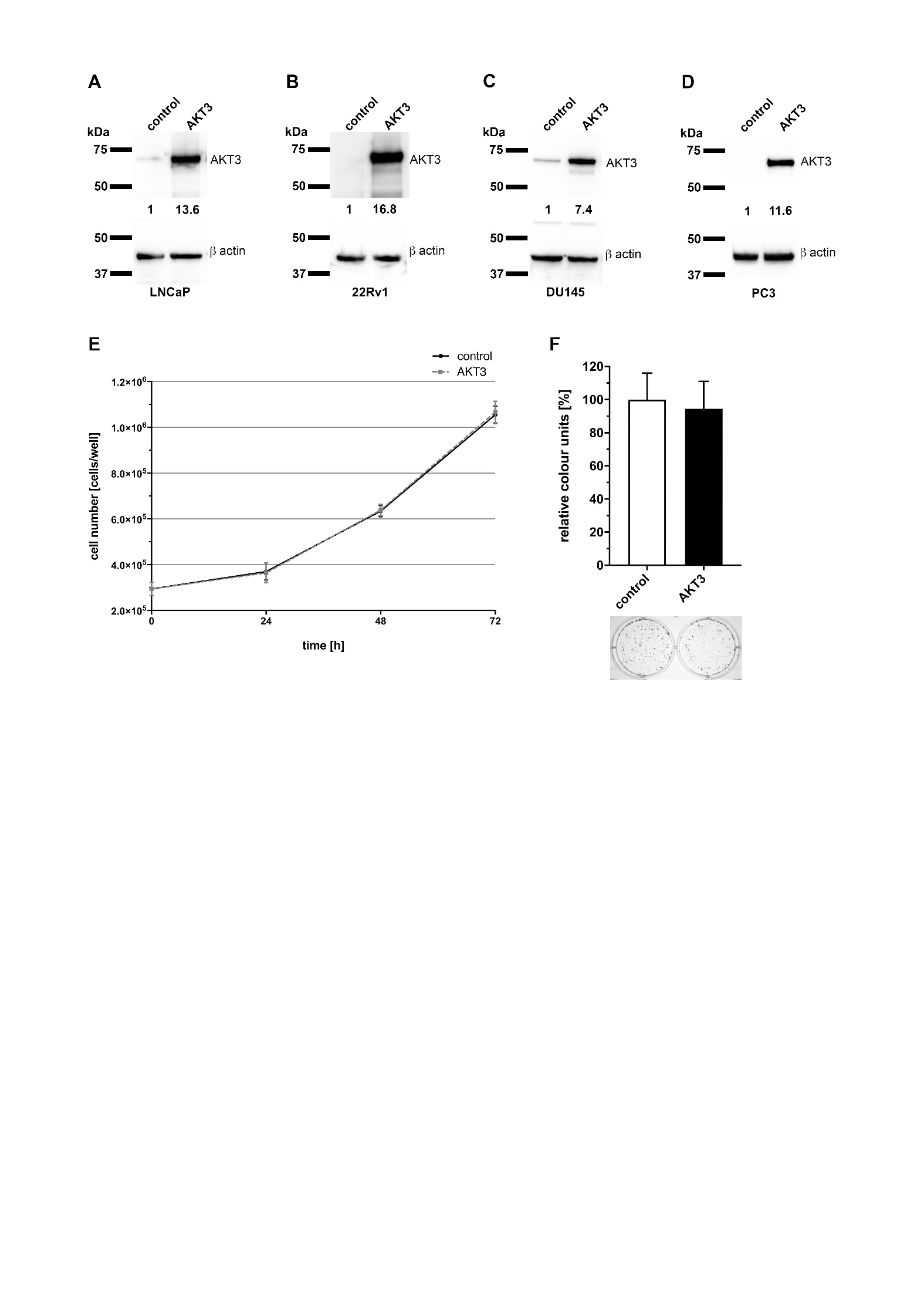


**Figure S3.** Expression of AKT3 after transfection of PCa cells with AKT3 expression vector was validated on protein level by western blot analysis with anti-AKT3 mAb using β-actin as loading control (**A–D**). The results show increased AKT3 protein expression in LNCaP cells 13.6-fold (**A**), 22Rv1 cells 16.8-fold (**B**), DU145 cells 7.4-fold (**C**) and PC3 cells 11.6-fold (**D**). Effects of AKT3 expression on cellular behavior. Effects of AKT3 expression on cell growth and colony formation on LNCaP cells were analyzed after transfection of LNCaP cells with control or AKT3 expression plasmid. Cell number was determined every 24 h after transfection in four independent experiments (**E**). Colony forming ability was evaluated 14 days after transfection, by fixing and staining colonies with crystal violet and densitometrical analysis. Upper graph shows the mean ± SEM of four independent experiments, lower picture depicts one representative result (**F**). AKT3 induction has no impact on proliferation or colony forming capacity of LNCaP cells.

**Table S1.** Oligonucleotides sequences.

| **Application** | **Gene** | **Sequence** |
| --- | --- | --- |
| qRT-PCR | AKT3 | F: 5'-TTGCTTTCAGGGCTCTTGAT-3' |
|  |  | R: 5'-CATAATTTCTTTTGCATCATCTGG-3' |
|  | 18S rRNA | F: 5'-CTACCACATCCAAGGAAGCA-3' |
|  |  | R: 5'-TTTTTCGTCACTACCTCCCCG-3' |
| cloning | AKT3 3‘UTR | F: 5'-GGACTAGTCTGGACATCACCAGTCCTAGC-3' |
|  |  | R: 5'-CGAGCTCGCCCTTGGCATGCATAGTTGG-3' |
|  | AKT3 cds | F: 5’-CGGCTAGCGCCGCCACCATGAGCGATGTTACCATTG-3’ |
|  |  | R: 5’-CGGAATTCTTATTCTCGTCCACTTGCAGA-3’ |
| site directed mutagenesis | AKT3 mutated 3‘UTR | F: 5'-GCTGATGAGAATCATCGTCGACTTCTCCTCTGTTAAA-3' |
|  |  | R: 5'-TTTAACAGAGGAGAAGTCGACGATGATTCTCATCAGC-3' |

Abbreviations: cds, coding sequence.

**Table S2.** Antibodies.

| **Antibody** | **Host** | **Conjugate** | **Clone** | **Company** |
| --- | --- | --- | --- | --- |
| anti-CHGA | rabbit | - | polyclonal | (ab15160) Abcam, Cambridge, UK |
| anti-SYP | rabbit | - | clone D8F6H | Cell Signaling, Frankfurt am Main, Germany |
| anti-AKT3 | mouse | - | clone L47B1 |  |
| anti-FOXO3A | rabbit | - | clone D19A7 |  |
| anti-phospho-FOXO3A (Ser253) | rabbit | - | polyclonal |  |
| anti-GSK3α/β | rabbit | - | clone D75D3 |  |
| anti-phospho-GSK3α/β (Ser21/9) | rabbit | - | clone D17D2 |  |
| anti-mouse IgG | goat | HRP | polyclonal | Pierce™ Thermo Fisher, Oberhausen, Germany |
| anti-rabbit IgG | goat | HRP | clone 31460 |  |
| anti-β-actin | mouse | HRP | clone AC15 | Sigma-Aldrich, Hamburg, Germany |
| anti-rabbit IgG | swine | biotin | polyclonal | DAKO, Agilent Technologies, Waldbronn, Germany |
| anti-mouse IgG | goat | Alexa 488 | polyclonal | Dianova, Hamburg, Germany |
