# Supplementary material for: Increased Expression of AKT3 in Neuroendocrine Differentiated Prostate Cancer Cells Alters the Response Towards Anti-Androgen Treatment
Source: Cancers (Basel). 2021 Feb 2;13(3):578. doi: 10.3390/cancers13030578 (PMC7867287; doi:10.3390/cancers13030578)

Figure 1B

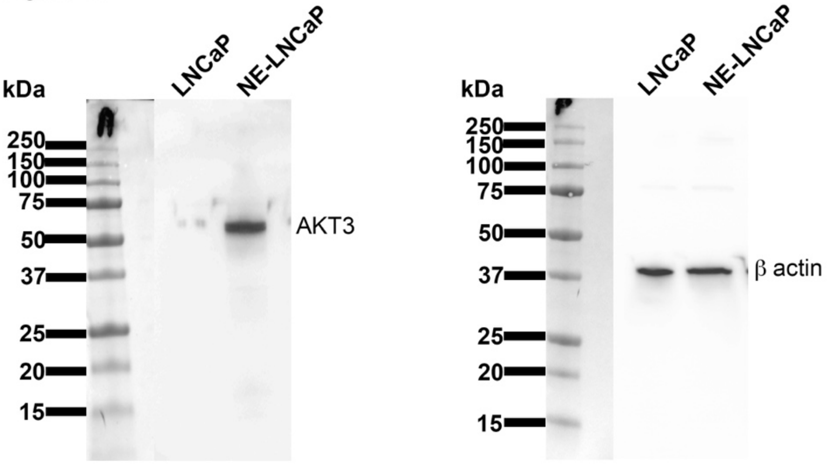

Figure 1D

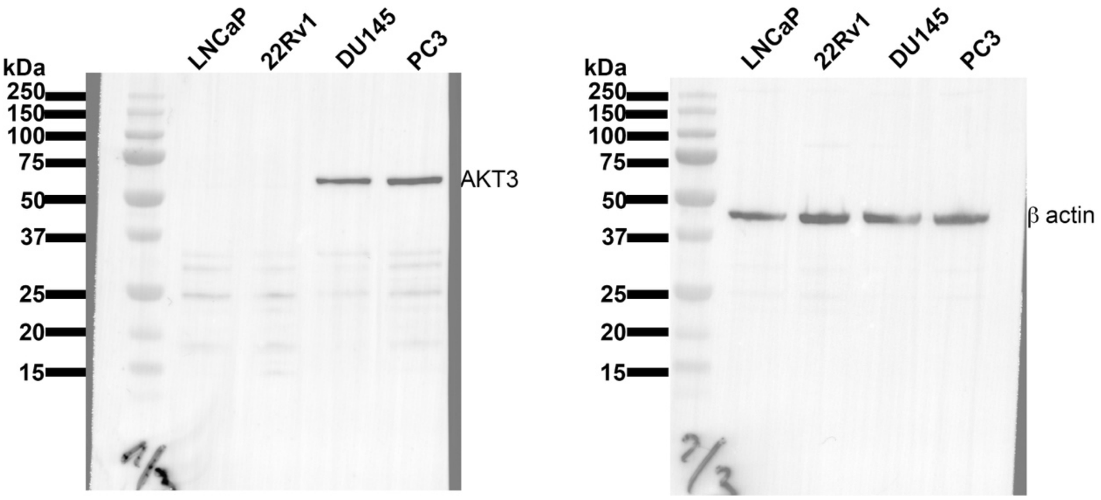

Figure 1E LNCaP

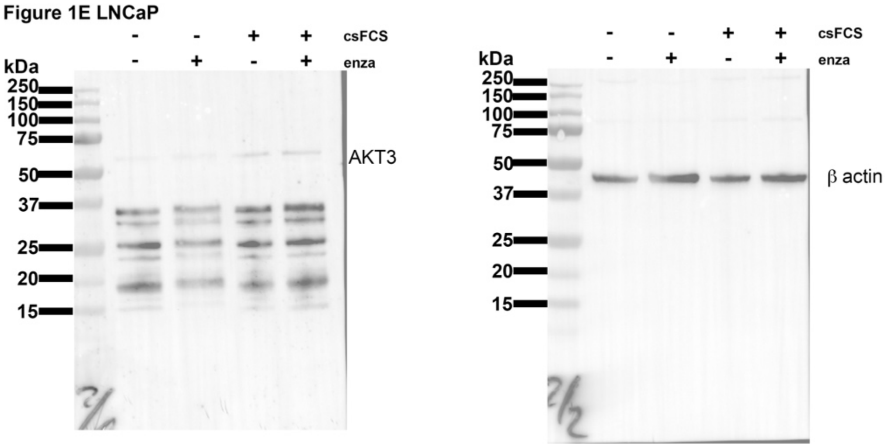

Figure 1F 22Rv1

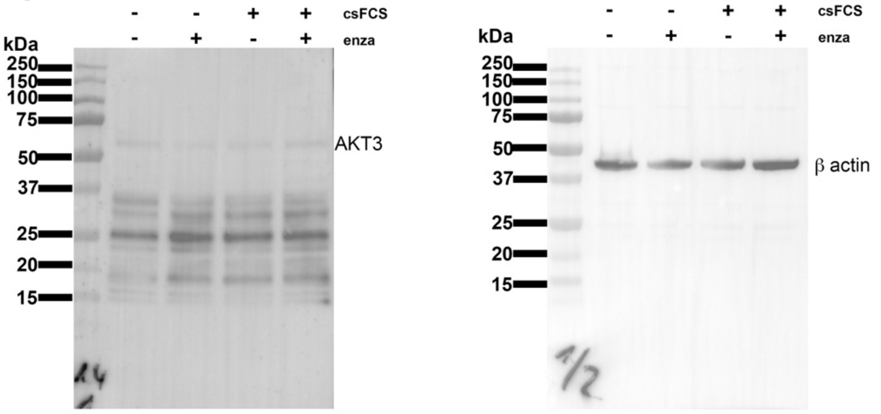

Figure 1F DU145

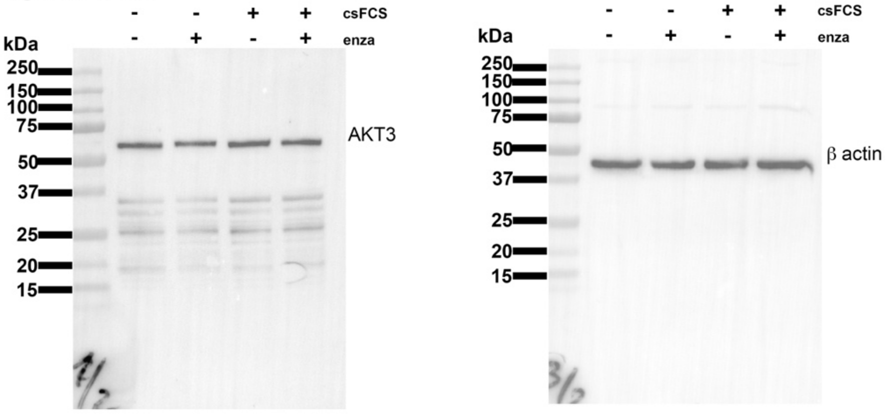

Figure 1F PC3

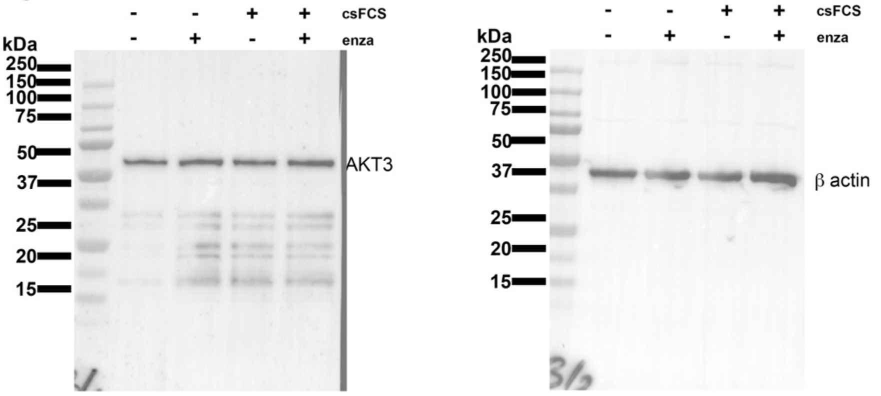

Figure 4A

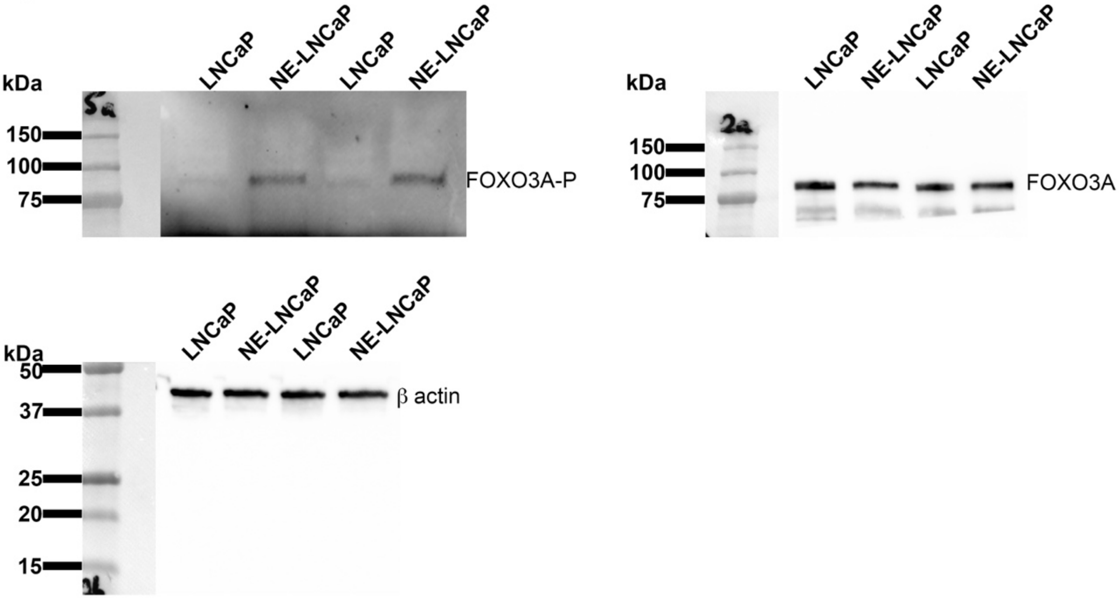

Figure 4C

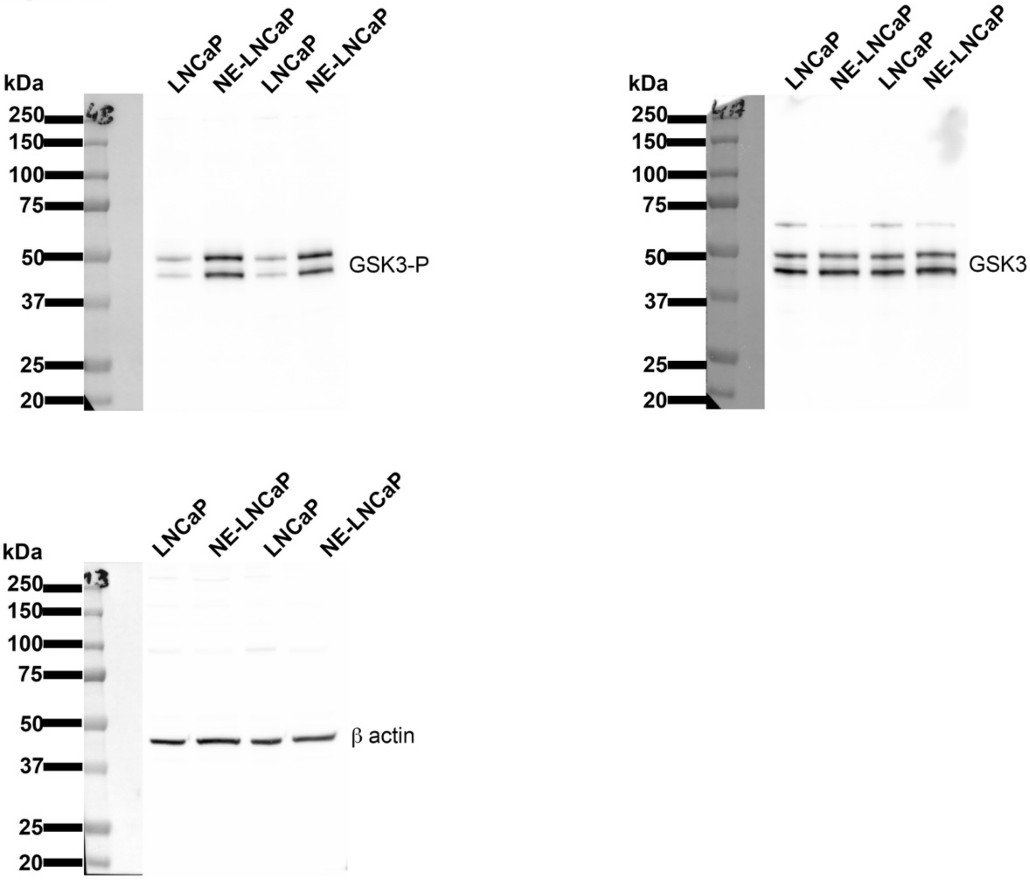

Figure 6E

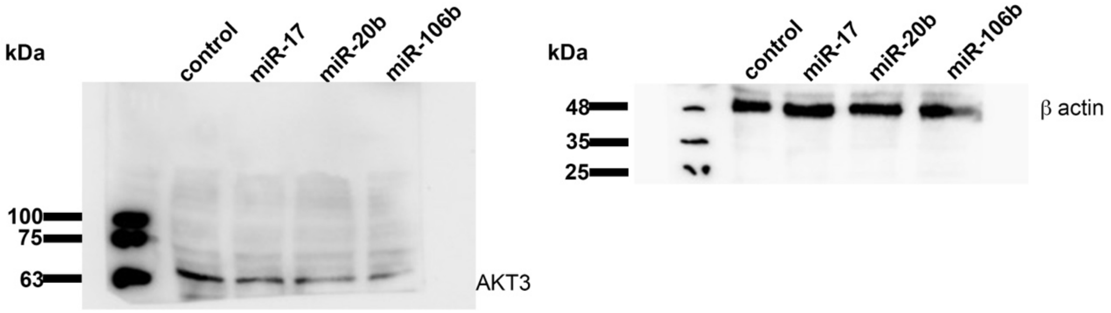

Figure S3A LNCaP

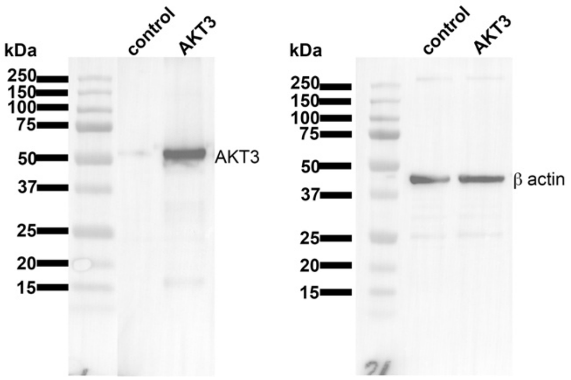

Figure S3B 22Rv1

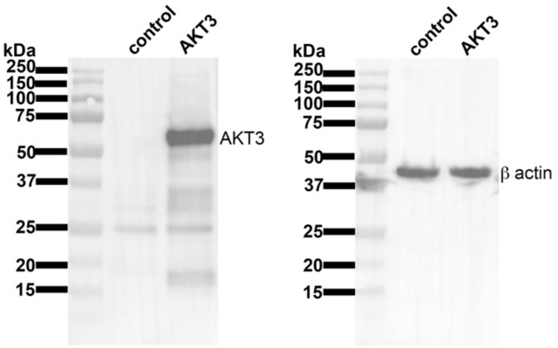

Figure S3C DU145

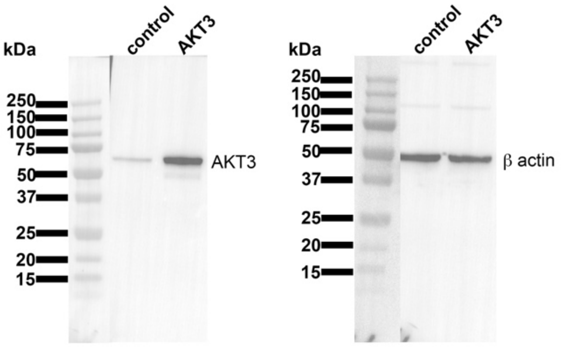

Figure S3D PC3

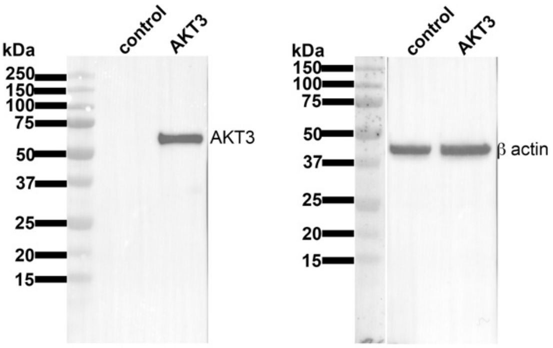

Supplement: Supplementary file 1 [file cancers-13-00578-s001.zip › cancers-1085417-supplementary-update/Figure S4 Uncropped Western blot images .pdf]
